# Cytokine alterations in CSF and serum samples of patients with a first episode of schizophrenia: results and methodological considerations

**DOI:** 10.1007/s00406-023-01569-y

**Published:** 2023-02-11

**Authors:** Deepti Singh, Paul C. Guest, Henrik Dobrowolny, Tino Fischbach, Gabriela Meyer-Lotz, Carolin Breitling-Ziegler, Aiden Haghikia, Stefan Vielhaber, Johann Steiner

**Affiliations:** 1 Department of Psychiatry, Otto-von-Guericke-University, Leipziger Str. 44, 39120 Magdeburg, Germany; 2 Laboratory of Translational Psychiatry, Otto-von-Guericke-University, Magdeburg, Germany; 3 Laboratory of Neuroproteomics, Department of Biochemistry and Tissue Biology, Institute of Biology, University of Campinas (UNICAMP), Campinas, Brazil; 4 Department of Child and Adolescent Psychiatry and Psychotherapy, Otto-von-Guericke-University, Magdeburg, Germany; 5 Department of Neurology, Otto-von-Guericke-University, Magdeburg, Germany; 6 Center for Behavioral Brain Sciences (CBBS), Magdeburg, Germany; 7 Center for Health and Medical Prevention (CHaMP), Magdeburg, Germany; 8 German Center for Mental Health (DZP), Center for Intervention and Research On Adaptive and Maladaptive Brain Circuits Underlying, Mental Health (C-I-R-C), Halle-Jena-Magdeburg, Germany

**Keywords:** First-episode psychosis, Schizophrenia, Serum, cerebrospinal fluid, CSF, Cytokines, Inflammation

## Abstract

We determined cytokine levels in paired serum/CSF samples from first-episode schizophrenia (FES) participants (*n* = 20) and controls (*n* = 21) using a 13-plex immunoassay. Applying strictly-determined detection limits, 12 cytokines were found in serum and two in CSF. Higher serum MCP-1 levels (*p* = 0.007) were present in FES versus controls, which correlated with serum IgG (*R* = − 0.750; *p* = 0.013). Finally, IL-18 levels correlated with body weight in FES (*R* = 0.691; *p* = 0.041). This study demonstrates potential limitations in the sensitivity of multiplex cytokine assays for CSF studies in mental disorders and suggests that some published studies in this area should be re-evaluated.

**Supplementary Information:**

The online version contains supplementary material available at 10.1007/s00406-023-01569-y.

## Introduction

Increasing evidence suggests that neuroinflammation contributes to the pathogenesis of psychiatric disorders, including schizophrenia [1–6]. As many psychiatric disorders are thought of as systemic diseases, it has proved useful to integrate findings from multiple biomarker sources, such as cerebrospinal fluid (CSF) and blood [7–10], and by using multiplex immunoassays [11–13] to provide a more holistic picture of health status. However, only a few studies have applied such approaches to investigate inflammation-related biomarkers in both media in first-episode schizophrenia (FES) [6, 11].

Since multiplex immunoassays can show low sensitivity and poor correlation with corresponding singleplex methods [14, 15], it is important to characterise the performance of these platforms in clinical/laboratory-based studies. The limit of detection (LOD) defines the smallest concentration of an analyte that can be measured [16]. Although critical for discriminating between the presence or absence of low abundance analytes such as cytokines, few studies have reported that this was done correctly. This is especially true for cytokine measurements in CSF, where many of these molecules are present at < 10 pg/mL concentrations. However, most multiplex immunoassay studies have reported low concentrations of specific cytokines in CSF in mental disorders, without correctly determining the LOD [6, 17–21].

Although the companies who developed these assays may claim dynamic ranges with low LODs, none are capable of accurately measuring concentrations that give readings in the range of blank samples. Threshold levels of analytes must be present to produce signals that can be distinguished above this noise [16, 22]. We suggest adherence to the Clinical and Laboratory Standards Institute (CLSI) guideline for accurately determining the limit of the blank (LOB) and LOD to increase confidence in the results [16, 22]. Here, we carried out multiplex immunoassay analyses using the most robust assays from a 13-plex immunoassay panel to identify differences in cytokine levels in serum and CSF from FES patients and controls, following the CLSI guideline in determining the LOB and LOD for each assay.

## Materials and methods

### Samples

The study was performed according to German laws, the Declaration of Helsinki, and local institutional review board guidelines. Participants gave written informed consent. CSF and sera were obtained from 20 FES in-patients diagnosed according to ICD10 and AWMF-S3 guidelines [23] for whom routine differential diagnostic lumbar- and veni-puncture had been performed (Supplementary Table ST1). Samples were collected 8.0 days (median) after admission with acute psychosis. Psychopathology was assessed using the Positive and Negative Syndrome Scale (PANSS) [24]. Exclusion criteria: (a) immunological concomitant diseases, recent/current infections, trauma/systemic diseases and substance abuse, (b) treatment with cortisone or other immunosuppressive/modulating substances. Administered antipsychotics were converted to chlorpromazine (CPZ) units for statistical purposes [25].

Controls (*n* = 21) had headache (*n* = 3), pseudotumor cerebri (*n* = 3) or initially unclear neurological symptoms (*n* = 15) with no history of psychiatric disorders and underwent lumbar puncture to rule out subarachnoid hemorrhage, infectious or autoimmune central nervous system disease. They were matched for age, gender and body mass index. Routine CSF parameters were within the normal range and showed no significant group differences.

After an investigation of 36 clinical parameters (Supplementary Table ST2, column B), CSF and sera were centrifuged and the supernatants stored at − 80 °C.

### Cytokine analysis

Concentrations of 13 cytokines [interferon (IFN)-α2, IFN-γ, interleukin (IL)-1β, IL-6, IL-8, IL-10, IL-12p70, IL-17A, IL-18, IL-23, IL-33, monocyte chemoattractant protein (MCP)-1 and tumor necrosis factor (TNF)-α] were determined in triplicate using the LEGENDplex™ Human Inflammation Panel 1 (BioVendor; Brno, Czechia) according to manufacturer instructions. This is a fluorescence–coded microsphere-based multiplex immuoassay for the detection and quantitation of analytes by flow cytometry. Sera were diluted twofold and CSF tested undiluted. Standard curves of each cytokine were analysed in duplicate on two separate occasions. The LOB and LOD were determined for each assay according to CLSI guidelines [16, 22], using the formulas below:

$$LoB\, = \,mean_{blank} \, + \,1.645\,\left( {SD_{blank} } \right).$$

$$LoD\, = \,LoB\, + \,1.645\,\left( {SD_{lowest\,concentration\,standard} } \right).$$

For statistical analysis, we used only those cytokines for which at least 2 of the 3 replicates and > 50% of samples gave readings above the LOD.

### Statistical analysis

Data were analyzed using R (v4.0.5). Chi-square tests were performed to calculate group differences regarding gender and smoking. Corrected PANSS scores were derived by subtraction of minimum from raw scores [26]. Most data were not normally distributed as indicated by Shapiro–Wilk tests. Thus, group differences of continuous variables were calculated by Mann–Whitney *U* tests. MCP-1 serum levels (*p* < 0.001) were significantly higher in smokers. Therefore, diagnosis-dependent differences in MCP-1 were calculated by ART (analysis of variance using an aligned rank transformation of the data) with the covariate smoking. Due to the exploratory nature of the study, group statistics were not corrected for multiple comparisons.

We used Spearman rank tests with false discovery rate (FDR)-corrected *p*-values (*q*-values) to identify correlations of cytokines with routine blood/CSF, demographic and clinical parameters. Cliff’s delta (δ) was used to assess effect sizes (δ ≥ 0.147 = small, δ ≥ 0.330 = medium, δ ≥ 0.474 = large) [27]. All statistical tests were two-tailed with *p* < 0.05 considered significant.

## Results and discussion

### Determination of valid cytokine assays

According to our criteria, 12 out of 13 cytokine assays could be measured in serum samples with > 50% giving readings above the LOD (Table 1). The excluded cytokine was IL1β, for which only 6 out of the 21 control and 3 of the 20 FES samples had values above the LOD. In contrast to the serum results, only two, namely MCP-1 and IL-8, could be measured in CSF.

**Table 1:** Valid cytokine assays

Cytokine	Diagnosis	CSF			Serum		
		Valid	Not valid	% Valid	Valid	Not vallid	% Valid
IFN-α2	Cont	0	21	0	20	1	**95.2**
IFN-α2	FES	0	20	0	20	0	**100**
IFN-γ	Cont	0	21	0	13	8	**61.9**
IFN-γ	FES	0	20	0	14	6	**70**
IL-10	Cont	0	21	0	20	1	**95.2**
IL-10	FES	2	18	10	20	0	**100**
IL-12p70	Cont	0	21	0	17	4	**81**
IL-12p70	FES	0	20	0	17	3	**85**
IL-17A	Cont	0	21	0	19	2	**90**
IL-17A	FES	1	19	5	19	1	**95**
IL-18	Cont	2	19	10	21	0	**100**
IL-18	FES	3	17	15	20	0	**100**
IL-1β	Cont	0	21	0	6	15	29
IL-1β	FES	0	20	0	2	18	10
IL-23	Cont	0	21	0	18	3	**86**
IL-23	FES	0	20	0	17	3	**85**
IL-33	Cont	0	21	0	19	2	**90.5**
IL-33	FES	0	20	0	20	0	**100**
IL-6	Cont	7	14	33.3	20	1	**95.2**
IL-6	FES	12	8	60	17	3	**85**
IL-8	Cont	20	1	**95.2**	19	2	**90.48**
IL-8	FES	20	0	**100**	20	0	**100**
MCP-1	Cont	21	0	**100**	20	1	**95**
MCP-1	FES	20	0	**100**	20	0	**100**
TNF-α	Cont	0	21	0	16	5	**76.2**
TNF-α	FES	0	20	0	15	5	**75**

Cytokine assays for which at least 2 out of the 3 replicates and > 50% of the samples gave readings above the LOD are indicated for CSF and serum control (CONT) and FES samples

The assays which passed these criteria are indicated in bold font

### Diagnosis-specific differences in cytokine levels

Neither of the two measurable CSF cytokines showed significant differences in concentrations between the groups (Table 2). The use of the current stringent approach might explain why we did not find some of the cytokine increases reported in a previous meta-analysis [6]. For the serum analysis, the levels of MCP-1 were significantly higher with a large effect size in FES (*n* = 20) [327.2 (225; 463.8) pg/mL] compared to controls (*n* = 19) [220.0 (108.5; 265.9) pg/mL, *p* = 0.007, *δ* = 0.495]. This was confirmed by ART with the covariate smoking [*p* = 0.024, *δ* = 0.479].

**Table 2:** Cytokine levels in serum and CSF from FES patients and controls

Cytokine	Cont [mean (Q1;Q2;*n*)]	FES [ean (Q1;Q2;*n*)]	*U* test	Cliff’s delta
Serum				
IFN-α2	9.213 (5.928;23.863;20)	11.46 (5.52;29.21;20)	0.698	− 0.075
IFN-γ	9.981 (7.183;30.639;13)	15.37 (5.56;40.08;14)	0.616	− 0.121
IL-10	13.36 (7.19;33.38;20)	21.32 (13.02;39.67;20)	0.218	− 0.230
IL-12p70	13.02 (9.95;39.49;17)	14.36 (8.32;23.80;17)	0.730	0.073
IL-17A	4.854 (2.895;13.419;19)	3.731 (2.997;6.110;19)	0.201	0.247
IL-18	345.3 (242.1;417.3;21)	358.6 (240.0;565.3;20)	0.561	− 0.110
IL-23	47.84 (25.85;100.07;18	49.18 (35.71;79.19;17)	0.883	− 0.033
IL-33	369.7 (115.0;555.0;19)	306.9 (135.8;409.6;20)	0.380	0.168
IL-6	23.27 (14.27;70.30;20)	25.32 (14.13;63.47;17)	0.964	0.012
IL-8	130.5 (62.3;221.1;19)	76.46 (60.63;150.68;20)	0.396	0.163
MCP-1	220.0 (108.5;265.9;19)	327.2 (225.7;463.8;20)	**0.007**^**#**^	− 0.495
TNF-α	32.01 (15.29;228.23;16)	27.16 (18.34;62.01;15)	0.495	0.150
CSF				
IL-8	118.0 (65.0;144.6;20)	93.77 (67.78;107.23;20)	0.429	0.150
MCP-1	457.5 (401.8;595.7;21)	452.1 (393.8;568.9;20)	0.847	0.038
CSF/serum				
IL-8	1.031 (0.485;1.567;18)	1.117 (0.494;1.810;20)	0.393	0.167
MCP-1	1.934 (1.401;4.065;20)	1.285 (0.931;2.393;20)	**0.030**^**+**^	0.400

Mean (Q1;Q3) values are in pg/mL

Significant differences between FES and controls (Cont) are indicated in bold

^#^ART with covariate smoking: *p* = 0.024

^+^ART with covariate smoking: *p* = 0.114

### Correlation of cytokines with other parameters

#### Serum MCP-1

**Fig. 1:**
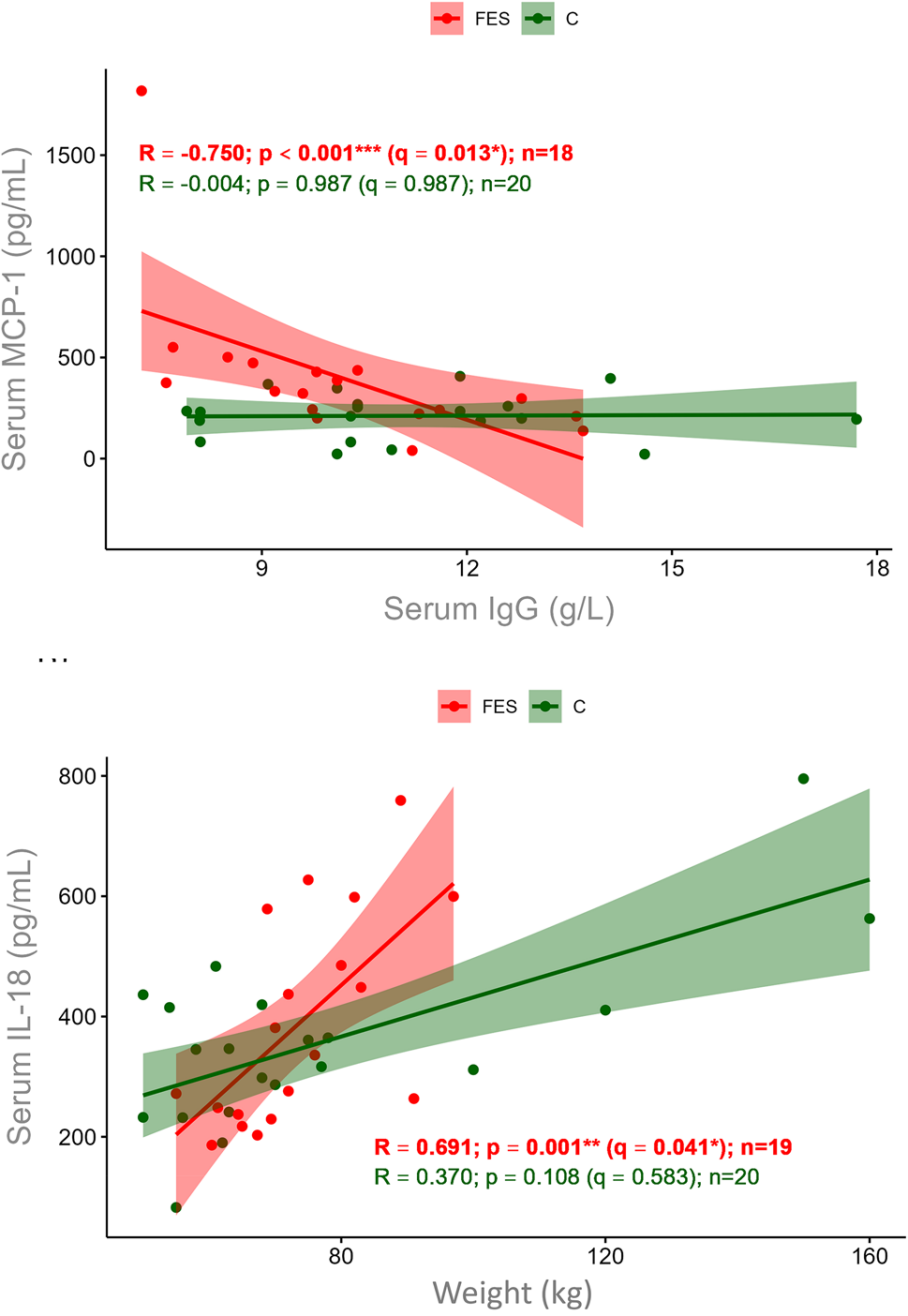
**A** Scatter plot showing Spearman rank correlation analysis of serum MCP-1 and IgG levels in FES (*n* = 18; red) and controls (C, *n* = 20; green). **B** Scatter plot showing Spearman rank correlation analysis of serum IL-18 levels with body weight (FES: *n* = 19, red; C:* n* = 20, green)

Of the 36 parameters determined (Supplementary Table ST2), there were no correlations with serum-MCP-1 apart from a significant negative correlation with serum IgG in FES (*R* = − 750; *p* < 0.001; *q* = 0.013) compared to controls (*R* = − 0.004; *p* = 0.987; *q* = 0.987) (Fig. 1A, Supplementary Table ST2). This supports previous findings of immunodeficiency-like and inflammatory phenotypes in schizophrenia [28–32]. Also, the finding of increased levels of MCP-1 in FES is consistent with previous studies which showed that schizophrenia patients have elevated serum MCP-1 levels in association with metabolic syndrome [33]. Although these physiological changes can be a side effect of antipsychotics, there have also been reports of such metabolic dysfunctions in first-onset patients prior to receiving medication [34]. Of note, serum MCP-1 levels did not correlate with PANSS scores (Supplementary Table ST3), CPZ units (*R* = − 0.154, *p* = 0.741, *q* = 0.919), albumin quotient (*R* = 0.579, *p* = 0.012; *q* = 0.169) or IgG index (*R* = 0.155, *p* = 0.538; *q* = 0.848).

#### Serum IL-18

Although serum IL-18 levels showed no significant differences between groups (*p* = 0.561; *U* test), IL-18 was significantly correlated with body weight (*R* = 0.691; *p* < 0.001; *q* = 0.041) in FES but not controls (*R* = 0.370; *p* = 0.108; *q* = 0.583) (Fig. 1B, Supplementary Table ST1). This is consistent with previous studies showing that, like MCP-1, IL-18 is linked to metabolic disorders including diabetes and insulin resistance [35–40] (Supplementary Table ST2).

#### Other cytokines

No other cytokines were correlated with the other measured parameters, disease duration or CPZ after FDR correction (q-values, Supplementary Tables ST2, ST4).

### Limitations

This study was limited as we only detected 2 out of 13 cytokines in CSF samples, obviating comparisons with cytokine readings in serum samples. Also, the small sample size of this exploratory investigation necessitates validation in larger cohorts. Regarding the biological findings, we did not assess the potential links of MCP-1 and IL-18 with metabolism-related disturbances as no measures of insulin resistance or visceral fat accumulation were available. Finally, although we controlled for the potential effects of antipsychotic medication, disease duration and smoking, we could not account for other influences like nutrition and sleep, and the use of headache patients as controls might bias the results as inflammatory causes may play a role in different forms of headache.

### Conclusions

To our knowledge, this is the first multiplex cytokine immunoassay study of paired serum/CSF samples from FES patients and controls using criteria based on strictly determined LODs. The main advantage of using the CLSI guideline is a reduction of false positives as this approach takes into account the variance of both the blank and lowest concentration samples^1^. The potential disadvantages include the possibility of not detecting low-concentration cytokines close to these variable regions. Using this stringent approach, we detected 12 cytokines in serum and only two in CSF using a 13-plex panel. The undetectable cytokine assays all gave signals that were indistinguishable from the blank readings. This revealed potential limitations in the sensitivity of multiplex cytokine assays of CSF. This calls attention to the need for more sensitive assays that can be used to obtain reliable readings in CSF, such as gold nanoparticle immuno-PCR [41, 42] and single molecule arrays [43, 44]. Both methods can provide sensitivities approximately 100- to 1000-fold greater than conventional multiplexed immunoassay systems.

## Supplementary Information

Below is the link to the electronic supplementary material.

Supplementary file1 (DOCX 15 KB)

Supplementary file2 (XLSX 51 KB)

Supplementary file3 (DOCX 12 KB)

Supplementary file4 (DOCX 265 KB)

## Data Availability

The data that support the findings of this study are available from the corresponding author upon reasonable request.

## Footnotes

1 Other methods like bead count/median fluorescence intensity approaches do not account for variance.
