# Supplementary material for: Cytokine alterations in CSF and serum samples of patients with a first episode of schizophrenia: results and methodological considerations
Source: Eur Arch Psychiatry Clin Neurosci. 2023 Feb 11;273(6):1387–93. doi: 10.1007/s00406-023-01569-y (PMC10449694; doi:10.1007/s00406-023-01569-y)
Supplement: Supplementary file 1 — Supplementary file1 (DOCX 15 KB) [file 406_2023_1569_MOESM1_ESM.docx]

**Supplementary Table 1** Demographic data, clinical and routine CSF characteristics of patients with FES and controls. Annotation: Categorical variables are presented as n-numbers; continuous variables are presented as median and interquartile range. CPZ, chlorpromazine units; LP, lumbar puncture.

| **Variables** | **Cont (n=21)** | **FES (n=20)** | **test** | **p-value** |
| --- | --- | --- | --- | --- |
| Age [years] | 35.0 (26.0;41.0) | 27.5 (25.0;46.8) | U-test | 0.539 |
| Gender [female/male] | f: 15 / m: 6 | f: 13 / m: 7 | Chi^2^-test | 0.915 |
| Smoking [yes/no] | yes: 3 / no: 17 | yes: 7 / no: 13 | Chi^2^-test | 0.273 |
| Body mass index [kg/m²] | 22.9 (20.0;27.0) | 23.6 (22.3;27.3) | U-test | 0.460 |
| Duration of psychosis [months] | - | 1.06 (0.49;3.14) | - |  |
| Time of LP [days after admission] | - | 8.00 (5.00;23.25) | - |  |
| Medication at time of LP [CPZ mg/day] | - | 200.0 (200.0;300.0) | - |  |
| PANSS-P corrected [score] | - | 8.00 (4.00;12.00) | - |  |
| PANSS-N corrected [score] | - | 7.00 (4.00;12.00) | - |  |
| PANSS-G corrected [score] | - | 15.00 (11.00;18.75) | - |  |
| PANSS total corrected [score] | - | 33.50 (19.75;38.25) | - |  |
| CSF cell count [n/µL] | 1 (0;2) | 1 (0;1) | U-test | 0.148 |
| CSF/serum albumin ratio [x10^-3^] | 4.87 (4.21;5.34) | 3.82 (3.17;5.66) | U-test | 0.223 |
| IgG-Index | 0.50 (0.50;0.55) | 0.50 (0.50;0.60) | U-test | 0.627 |
