# Supplementary material for: Cytokine alterations in CSF and serum samples of patients with a first episode of schizophrenia: results and methodological considerations
Source: Eur Arch Psychiatry Clin Neurosci. 2023 Feb 11;273(6):1387–93. doi: 10.1007/s00406-023-01569-y (PMC10449694; doi:10.1007/s00406-023-01569-y)
Supplement: Supplementary file 3 — Supplementary file3 (DOCX 12 KB) [file 406_2023_1569_MOESM3_ESM.docx]

**Supplementary Table ST3.** Spearman correlation analyses showing no significant association of serum MCP-1 levels with PANSS scores in FES patients (n=20).

| **PANSS scale** | **r** | **p** | **q** |
| --- | --- | --- | --- |
| Positive | -0.046 | 0.847 | 0.919 |
| Negative | -0.409 | 0.073 | 0.476 |
| General | -0.136 | 0.568 | 0.852 |
| Total | -0.209 | 0.377 | 0.817 |
