# Supplementary material for: Cytokine alterations in CSF and serum samples of patients with a first episode of schizophrenia: results and methodological considerations
Source: Eur Arch Psychiatry Clin Neurosci. 2023 Feb 11;273(6):1387–93. doi: 10.1007/s00406-023-01569-y (PMC10449694; doi:10.1007/s00406-023-01569-y)

**Supplementary Table ST4.** Spearman correlation analyses showing no significant association of cytokine measures and white blood cell counts with duration of illness or antipsychotic daily dose (in chlorpromazine units) in FES patients (n=20).


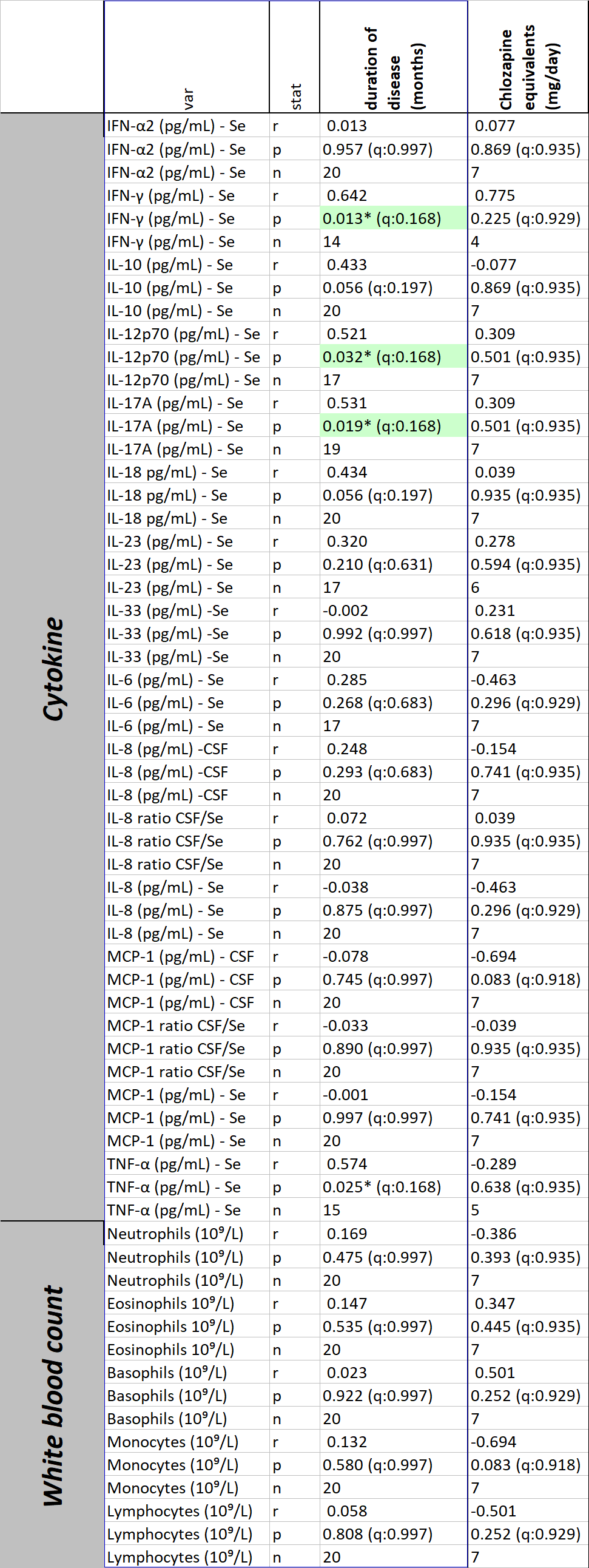

Supplement: Supplementary file 4 — Supplementary file4 (DOCX 265 KB) [file 406_2023_1569_MOESM4_ESM.docx]
